# Endovascular Stent Grafting for Iatrogenic Deep Femoral Artery Injury After Short Femoral Nailing: A Case Report With a Three-Year Follow-Up

**DOI:** 10.7759/cureus.89845

**Published:** 2025-08-11

**Authors:** Yuuki Mitani, Kazuhiro Ikeda, Takamasa Kudo, Sho Kohyama, Takaji Yanai

**Affiliations:** 1 Department of Orthopedic Surgery, Kikkoman General Hospital, Noda, JPN; 2 Department of Orthopedic Surgery, Institute of Medicine, University of Tsukuba, Tsukuba, JPN

**Keywords:** deep femoral artery injury, profunda femoris artery injury, pseudoaneurysm, short femoral nailing, stent grafting

## Abstract

Injury to the deep femoral artery (DFA) is a rare but potentially life-threatening complication of intertrochanteric femoral fracture surgery. We describe a case in which this complication occurred during drilling for the distal screw insertion of a short femoral nail. A 4 cm pseudoaneurysm was treated with stent grafting. Hemostasis was promptly achieved, and no recurrence of the aneurysm was observed over three years. The stent remained intact without fracture or migration. Although the stent lumen became nearly occluded during follow-up, the patient remained asymptomatic without signs of lower limb ischemia. This case highlights the utility of stent grafting for managing large pseudoaneurysms in DFA injury, where coil embolization may be unsuitable. In cases of isolated DFA injury, treatment selection should be guided by the clinical indication for pseudoaneurysm management, rather than the objective of preserving long-term arterial patency.

## Introduction

Injury to the deep femoral artery (DFA) is a rare but serious complication of intertrochanteric femoral fractures treated with a short femoral nailing (SFN), occurring in approximately 0.2-0.5% of cases [1]. DFA injury can be life-threatening since most patients with these fractures are elderly and often have compromised circulatory reserves [2]. Minimally invasive endovascular treatment has become the mainstay for managing DFA injuries, and in recent years, stent grafting has been reported as a means to achieve hemostasis while preserving arterial blood flow [3,4]. However, evidence regarding the long-term durability of stent grafts remains limited. This report presents a case of iatrogenic DFA injury treated with endovascular stent grafting. Stent grafting promptly controlled the severe hemorrhage, which had required 10 units of red blood cell transfusions during a 10-day waiting period before the procedure. The three-year follow-up offers insight into both the clinical utility and the limitations of this therapeutic approach.

## Case presentation

**Figure 1:**
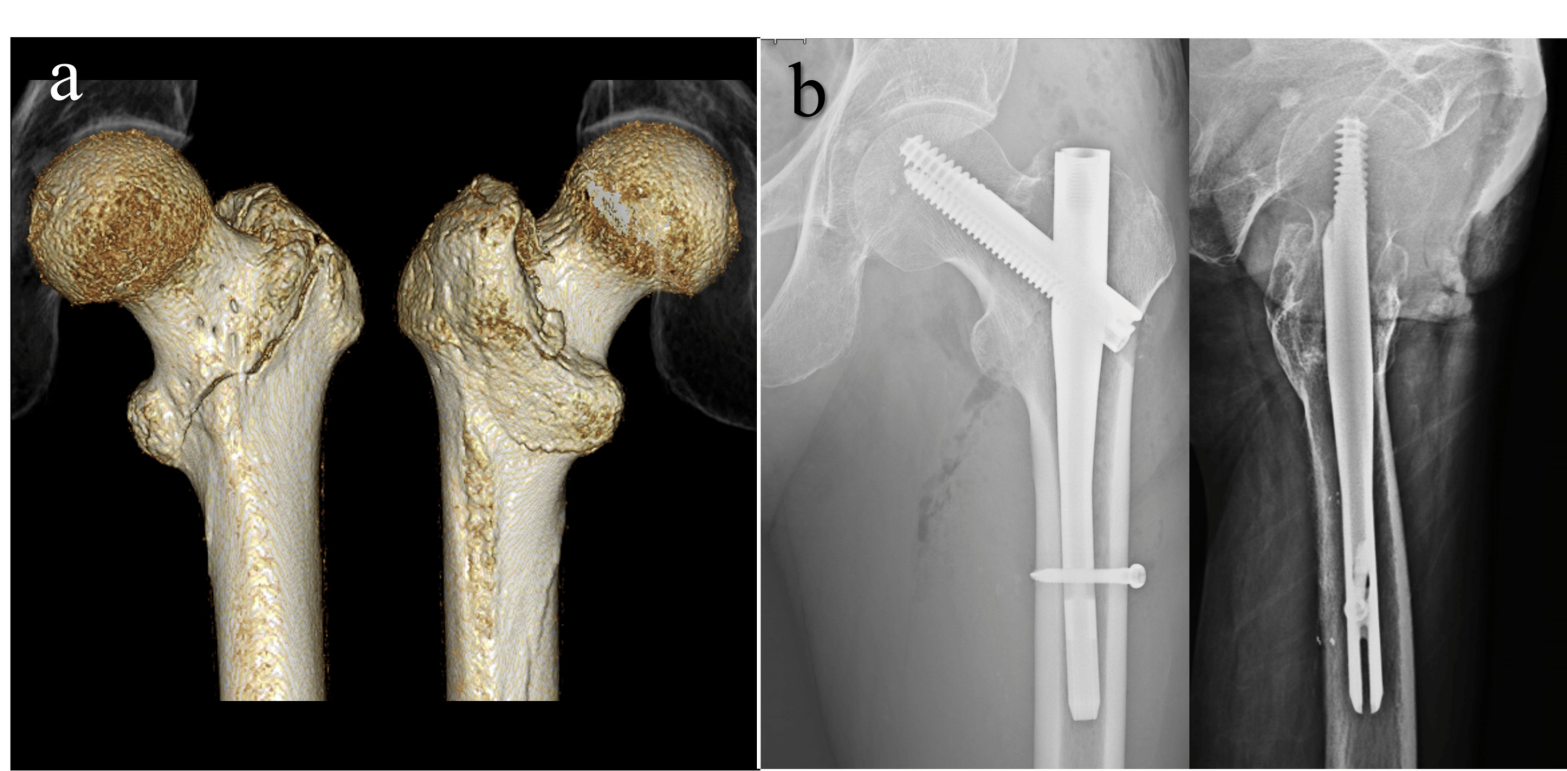
Pre- and postoperative imaging (a) Preoperative three-dimensional computed tomography (3D-CT) showing a nondisplaced intertrochanteric fracture of the left femur. (b) Postoperative plain radiographs after short intramedullary nailing

An 85-year-old man was brought to our emergency department after a fall, complaining of pain in the left hip (Table 1). Plain radiography and computed tomography (CT) revealed a nondisplaced two-part intertrochanteric fracture of the left femur (Figure 1). The patient underwent surgery the following day.

**Table 1:** Clinical characteristics of the patient BMI: body mass index

Parameters	Values
Age/sex	85/male
Presenting complaint	Left hip pain after a fall
Medical history	Chronic hepatitis C: treated with interferon
	Hemorrhagic duodenal ulcer: status postsubtotal gastrectomy
Medications	Ramelteon and suvorexant for insomnia management
Height/weight/BMI	162 cm/54 kg/20.7
Ambulation status	Independent before injury

Surgery

We positioned the patient on a traction table, placing the unaffected leg in the frog-leg position and adducting the affected leg against a perineal post. Without performing any reduction maneuver, we inserted an SFN (INTERTAN®, Smith & Nephew, United Kingdom; 125°, 10 mm × 180 mm) in situ. During insertion of the distal screw, we kept the affected leg adducted and carefully drilled through the far cortex. Intraoperative blood loss was 80 mL, and hemodynamics remained stable.

Postoperative course

**Figure 2:**
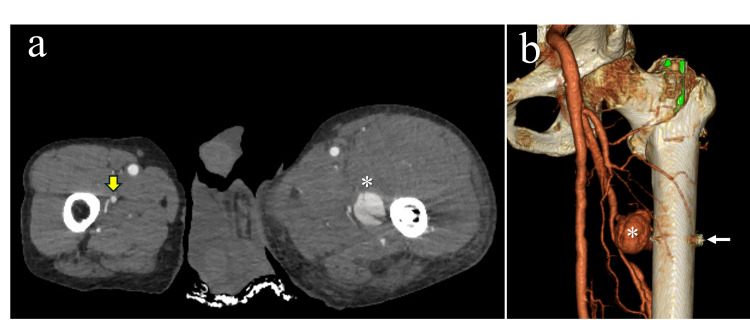
Postoperative contrast-enhanced computed tomography DFA: deep femoral artery (a) Axial image at the level of distal screw insertion. The affected thigh is swollen to approximately 1.5 times the transverse diameter of the contralateral side. A pseudoaneurysm with a false lumen is observed in the DFA (asterisk). The contralateral DFA lies 8 mm medial to the femur (thick yellow arrow). (b) Three-dimensional reconstruction showing a pseudoaneurysm (asterisk) at the same level as the distal screw insertion site (thin white arrow)

On postoperative day 1, the patient complained of pain in the medial thigh. There was significant femoral swelling, and his hemoglobin level had dropped from 12.7 mg/dL on admission to 8.0 mg/dL. On postoperative day 4, his hemoglobin had dropped further to 6.4 mg/dL, and he received a transfusion of two units of red blood cells. We suspected vascular injury based on marked femoral swelling, persistent medial thigh pain, and progressive anemia. Contrast-enhanced CT revealed a pseudoaneurysm measuring approximately 4 cm in diameter in the left DFA (Figure 2). The pseudoaneurysm was located at the level of the distal screw, consistent with vascular injury caused by intraoperative drilling. Considering the risk of pseudoaneurysm enlargement, we instructed the patient to remain on strict bed rest.

**Figure 3:**
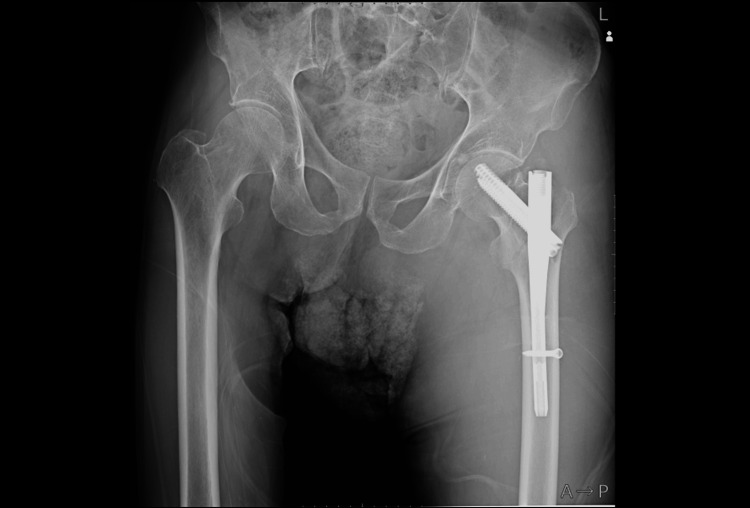
Plain radiograph on postoperative day 7 The affected thigh is markedly swollen

On postoperative day 5, hemoglobin remained low at 6.4 g/dL with poor improvement, which necessitated transfusion of four units of red blood cells. We consulted the cardiology department for endovascular treatment of the DFA injury, considering the patient’s advanced age and comorbidities. The cardiologist selected stent grafting as the preferred treatment, given the relatively large size of the pseudoaneurysm. The procedure was scheduled electively on postoperative day 10, as our institution had only one interventional cardiologist, and additional staffing arrangements were required. Swelling in the left thigh progressively worsened during this waiting period (Figure 3).

Percutaneous peripheral intervention (PPI) and long-term outcome

**Figure 4:**
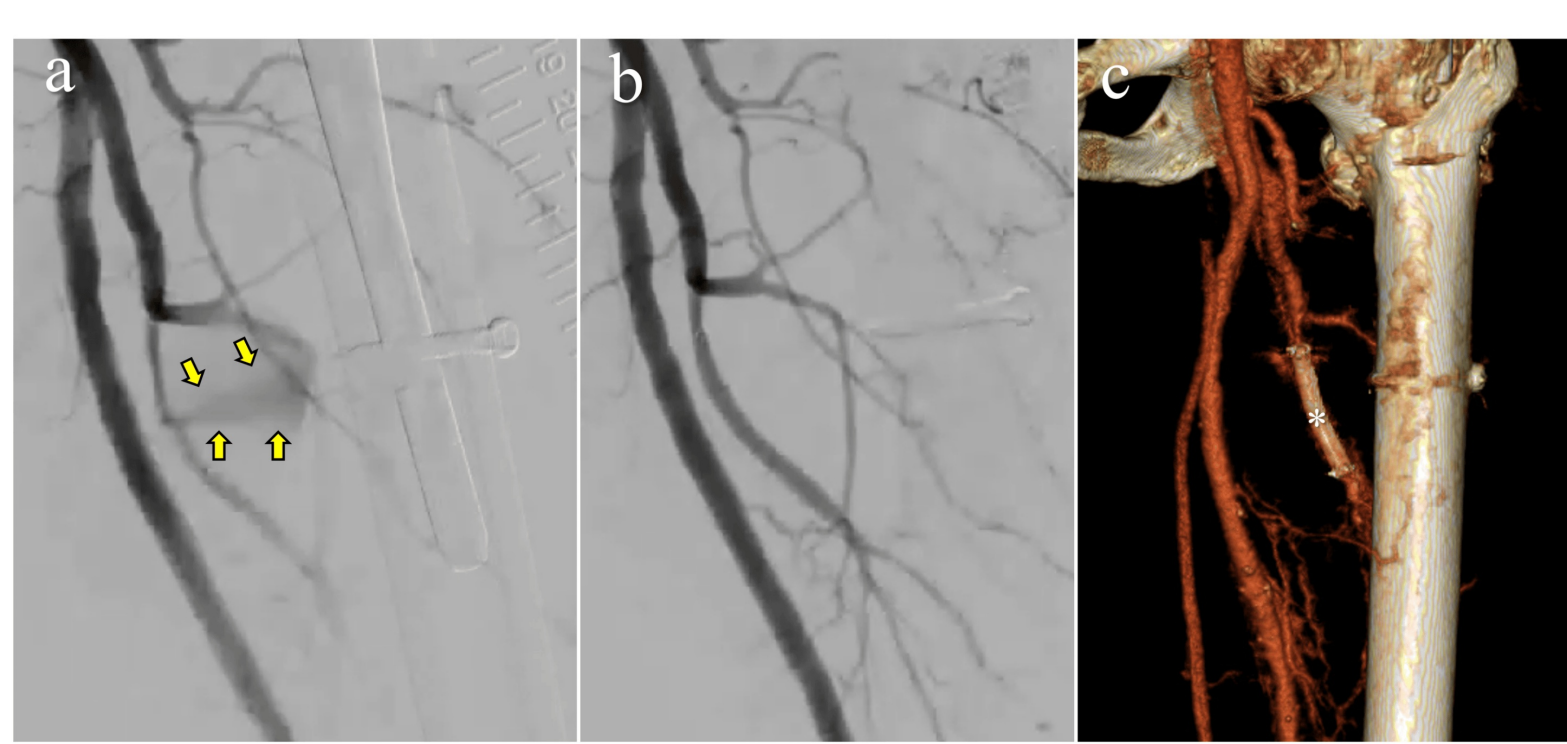
Angiographic findings before and after stent grafting (a) Digital subtraction angiography before treatment showing contrast extravasation from the deep femoral artery (yellow arrows). (b) Posttreatment angiography confirming successful hemostasis and restoration of blood flow. (c) Three-dimensional computed tomography angiography demonstrating the deployed stent graft (asterisk) in the deep femoral artery, with no visualization of the pseudoaneurysm

On postoperative day 10, hemoglobin dropped to 5.8 g/dL, and we transfused four units of red blood cells and six units of fresh frozen plasma. Subsequently, PPI was performed (Figure 4). A 6.0 mm × 50 mm VIABAHN® stent graft (W. L. Gore & Associates, Japan) was deployed in the main trunk of the left DFA, successfully excluding the pseudoaneurysm from circulation. From the following day, dual antiplatelet therapy with aspirin and clopidogrel was initiated and continued for one week. The patient was subsequently switched to single-agent therapy with rivaroxaban, which was discontinued after six months as the patient chose to discontinue cardiology follow-up.

From postoperative day 11, the thigh swelling gradually improved, and the patient's ambulatory ability steadily recovered. By week 6, the patient was walking independently with a walker, and by three months, with a T-cane. At one year, he was able to walk unassisted.

**Figure 5:**
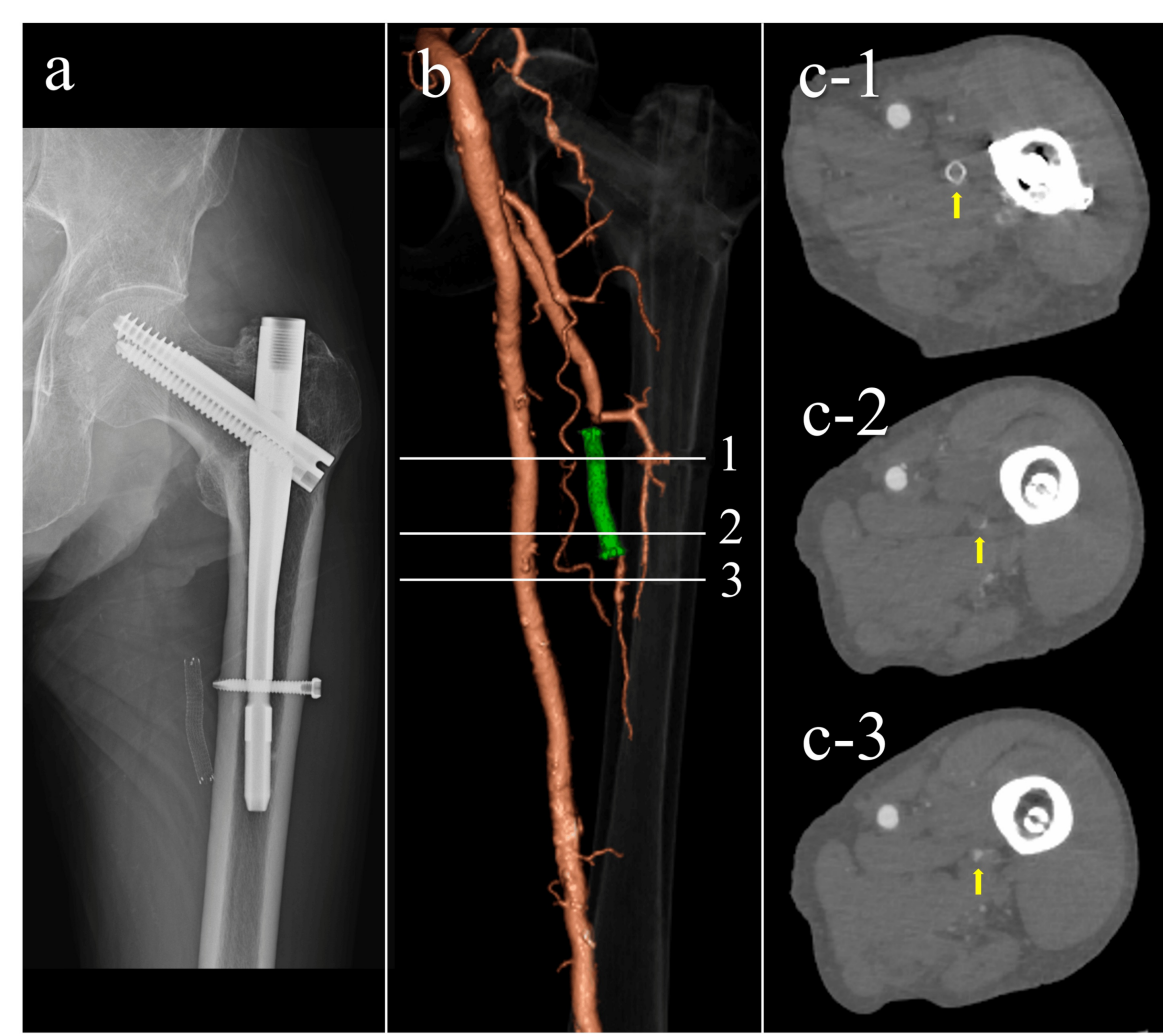
Follow-up imaging at three years postoperatively (a) Plain radiograph showing no fracture or migration of the stent. (b) Three-dimensional computed tomography angiography showing the deployed stent graft (green) in the deep femoral artery. A thin peripheral vessel is visible distal to the stent, indicating minimal preserved flow. Regions 1 to 3 correspond to the cross-sectional slices shown in c-1 to c-3. (c) Axial contrast-enhanced CT images at three levels (1 to 3 in b), demonstrating near-complete occlusion of the stent lumen (yellow arrows) without evidence of lower limb ischemia

At three years postoperatively, he remained ambulatory without assistance and reported no symptoms related to the affected limb. Plain radiographs showed no evidence of stent fracture or kinking. Contrast-enhanced CT revealed no recurrence of the pseudoaneurysm. Although the stent graft lumen was nearly occluded, 3D reconstructed images demonstrated minimal but persistent distal blood flow. As the patient exhibited no symptoms of lower limb ischemia, follow-up for the pseudoaneurysm and associated vascular lesion was concluded (Figure 5).

## Discussion

**Figure 6:**
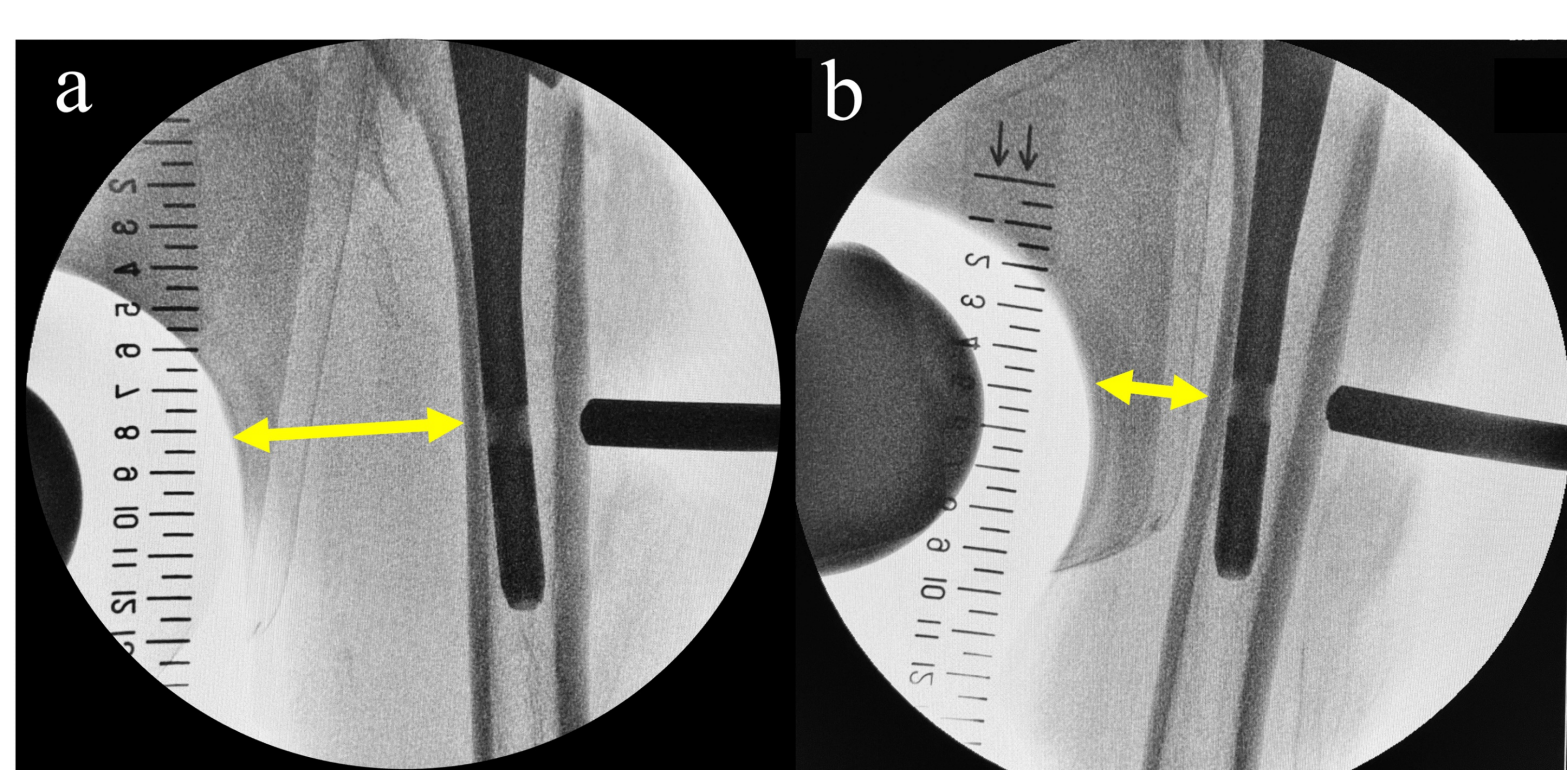
Intraoperative fluoroscopic images These images illustrate the effect of hip positioning on the medial shift of the femur. (a) With the hip in a neutral position, the medial cortex of the femur is relatively distant from the drill trajectory (yellow double-headed arrow). (b) With the hip in adduction, the medial cortex shifts closer to the drill path (yellow double-headed arrow), increasing the risk of vascular injury. Images are from a different patient and are provided for illustrative purposes

This case highlights a rare complication of DFA injury caused by drilling during distal screw insertion in SFN. It offers specific insights that may help prevent recurrence and guide appropriate treatment decisions. This injury to the DFA resulted from a combination of intraoperative limb positioning and patient-specific anatomical features. The DFA courses obliquely along the medial femur, running from the anterior proximal to the posterior distal aspect [5]. At the level of distal screw insertion of the SFN, it lies close to the medial cortex [5], a location that corresponds to the contact area of the perineal post. When the hip is adducted, the DFA is compressed medially and shifts closer to the femur, while its mobility is also reduced (Figure 6) [6,7]. In this case, limited subcutaneous fat over the medial thigh likely increased the risk of DFA compression by the perineal post. To reduce the risk of DFA injury during drilling for distal screw insertion, hip adduction should be released.

DFA injury following SFN is difficult to detect intraoperatively due to limited visualization of the medial femur [3,8]. Symptoms of DFA injury are typically nonspecific, including progressive swelling, pain, and unexplained anemia, often resulting in delayed diagnosis [9]. In this case, careful assessment of symptom severity and pain distribution facilitated early detection, despite their nonspecific nature. Further accumulation of cases is necessary to identify diagnostic cutoff values for clinical parameters.

This case demonstrated a favorable three-year outcome following stent grafting for DFA injury. Treatment options for DFA injury are broadly classified into surgical repair and endovascular intervention. Endovascular approaches have recently gained popularity as a minimally invasive treatment option [3,4,7-9]. Coil embolization is the mainstay of endovascular treatment for DFA injury and has been reported to achieve a technical success rate exceeding 90% [10]. However, it may be unsuitable for large pseudoaneurysms (>4 cm), wide-neck lesions (>1 cm), or cases requiring preservation of DFA perfusion [3,10-13]. In contrast, stent grafting can be applied even in such challenging cases, enabling rapid hemostasis by sealing the bleeding point within the pseudoaneurysm [11,14]. In our case, stent grafting achieved durable resolution of a large DFA pseudoaneurysm over three years. Notably, the device remained intact for three years despite being placed near a joint, where repeated mechanical stress could potentially cause fracture or migration [15]. This outcome supports the long-term utility of stent grafting for DFA injury.

While stent grafting is a viable option, its indication for isolated DFA injury should be carefully considered. The DFA has abundant collateral circulation, and its occlusion rarely results in lower limb ischemia [3,7-10]. At three years postoperatively, the stent in this case showed incomplete occlusion without any associated ischemic symptoms. Given the reported 78% one-year patency rate for stent grafts placed in the superficial femoral artery, a similar risk of occlusion may be expected in the DFA. Since stent grafting is more costly than coil embolization, treatment should primarily be selected based on the indication for pseudoaneurysm management, rather than on the preservation of DFA perfusion.

This report has several limitations. Hemodynamic evaluation was limited to the immediate postoperative and three-year follow-up points, with no intermediate assessment. In addition, the relationship between antithrombotic therapy and stent occlusion remains unclear.

## Conclusions

In this case, stent grafting for deep femoral artery injury achieved rapid hemostasis and complete exclusion of the pseudoaneurysm. Over three years of follow-up, no aneurysm recurrence or ischemic symptoms were observed, despite incomplete in-stent occlusion. These findings support the role of stent grafting as an effective option for achieving prompt bleeding control and definitive aneurysm exclusion, particularly in cases involving large or wide-neck pseudoaneurysms where coil embolization may be unsuitable. Further studies are needed to clarify the appropriate indications and long-term outcomes of this treatment through the accumulation of additional cases and prospective investigation.
